# Low-energy, high risk: unveiling the undertriage crisis in geriatric trauma

**DOI:** 10.1186/s13049-024-01285-z

**Published:** 2024-11-05

**Authors:** Adem Az

**Affiliations:** Department of Emergency Medicine, University of Health Sciences, Haseki Training and Research Hospital, Istanbul, Turkey

Dear Editor,

I read the recent study by Poncet et al. [1] with great interest. It offers a valuable contribution to geriatric trauma care, particularly in highlighting the challenges faced by elderly patients in receiving appropriate care. The study draws attention to disparities in trauma centre admissions and trauma team activation (TTA) for older patients, which is a crucial topic as the global population ages. However, I believe certain aspects of the study warrant further clarification and analysis. I respectfully offer the following queries and suggestions, which I hope will contribute to future research in this important area.

Firstly, the study only considers patients who were admitted to emergency services due to trauma, focusing on the rates of admission to the regional trauma centre and resuscitation room with TTA. However, it does not account for cases where patients requested emergency medical assistance but were never transported to the hospital due to undertriage. To draw more definitive conclusions about undertriage in elderly patients, it is crucial to analyse data from all emergency medical services (EMS) calls, including those who were not transported. Were there any patients triaged over the phone after calling EMS, or any that EMS teams evaluated at the scene but deemed unsuitable for transport? Without this, generalising undertriage based solely on patients admitted to the hospital could lead to misleading conclusions.

Secondly, the study noted that higher-energy trauma mechanisms, such as motor vehicle accidents and stabbings, were more prevalent in younger patients, as shown in Table 1 [1]. In contrast, low-energy trauma mechanisms, such as falls, were more common in older adults. As expected, patients with high-energy injuries were more likely to be transported to trauma centres. Therefore, regardless of BATT and MGAP scores, younger patients are more likely to be transported to trauma centres, whereas older patients with low-energy injuries may experience undertriage more frequently. Instead of directly comparing the entire young and older populations, comparisons within subgroups would provide clearer insights. For example, is there any findings that older adults are undertriaged more frequently than younger adults in motor vehicle accidents or falls? As such, the conclusion that older adults experience more undertriage could be adjusted to emphasise that even low-energy traumas, such as falls, can result in significant morbidity and mortality in older adults.

Lastly, I would like to address the absence of a direct comparison between trauma mechanisms in males and females. Demographic studies show that males are generally more likely to experience higher-energy trauma compared to females, which could explain the findings in Poncet et al.‘s study [2, 3]. However, the study lacks direct findings to support this difference. A comparison of trauma mechanisms by sex would have provided clearer insights into how the more frequent low-energy traumas in females contribute to higher undertriage rates. Including such an analysis would have helped to more comprehensively illustrate the sex differences in undertriage.

I sincerely appreciate the opportunity to provide feedback on this important study. Addressing these aspects would further enhance the study’s contribution to understanding trauma care for older patients.

Yours sincerely,

## Data Availability

No datasets were generated or analysed during the current study.
